# Cathepsin B-Overexpressed Tumor Cell Activatable Albumin-Binding Doxorubicin Prodrug for Cancer-Targeted Therapy

**DOI:** 10.3390/pharmaceutics14010083

**Published:** 2021-12-29

**Authors:** Hanhee Cho, Man Kyu Shim, Suah Yang, Sukyung Song, Yujeong Moon, Jinseong Kim, Youngro Byun, Cheol-Hee Ahn, Kwangmeyung Kim

**Affiliations:** 1 Biomedical Research Institute, Korea Institute of Science and Technology (KIST), Seoul 02792, Korea; ricky@kist.re.kr (H.C.); mks@kist.re.kr (M.K.S.); haehwan@kist.re.kr (S.Y.); t17192@kist.re.kr (S.S.); phoenix0310@kist.re.kr (Y.M.); 218843@kist.re.kr (J.K.); 2 Department of Materials Science and Engineering, Seoul National University, Seoul 08826, Korea; 3 KU-KIST Graduate School of Converging Science and Technology, Korea University, Seoul 02841, Korea; 4 Department of Biosystems & Biotechnology, Korea University, Seoul 02841, Korea; 5 Department of Bioengineering, Korea University, Seoul 02841, Korea; 6 Research Institute of Pharmaceutical Sciences, College of Pharmacy, Seoul National University, Seoul 08826, Korea; yrbyun@snu.ac.kr

**Keywords:** prodrug, albumin, drug delivery, targeted therapy, chemotherapy

## Abstract

Prodrugs are bioreversible medications that should undergo an enzymatic or chemical transformation in the tumor microenvironment to release active drugs, which improve cancer selectivity to reduce toxicities of anticancer drugs. However, such approaches have been challenged by poor therapeutic efficacy attributed to a short half-life and low tumor targeting. Herein, we propose cathepsin B-overexpressed tumor cell activatable albumin-binding doxorubicin prodrug, Al-ProD, that consists of a albumin-binding maleimide group, cathepsin B-cleavable peptide (FRRG), and doxorubicin. The Al-ProD binds to in situ albumin, and albumin-bound Al-ProD indicates high tumor accumulation with prolonged half-life, and selctively releases doxorubicin in cathepsin B-overexpressed tumor cells, inducing a potent antitumor efficacy. Concurrently, toxicity of Al-ProD toward normal tissues with innately low cathepsin B expression is significantly reduced by maintaining an inactive state, thereby increasing the safety of chemotherapy. This study offers a promising approach for effective and safe chemotherapy, which may open new avenues for drug design and translational medicine.

## 1. Introduction

Chemotherapy is still the first-line treatment option owing to its high sensitivity against wide range of cancers, but it is often accompanied by serious side effects attributed to a lack of cancer selectivity [1]. The risk of side effects by chemotherapy restricts drug dosage, which may limit the tumors from being exposed to sufficiently high drug concentrations, eventually leading to treatment failure [2]. Thus, many endeavors have been made to overcome these issues by improving the cancer selectivity of anticancer drugs to tumors. One of the promising approaches, prodrug, involves bioreversible medications that should undergo an enzymatic or chemical transformation in the tumor microenvironment to release active drugs, which greatly improve the cancer selectivity to reduce the off-target toxicity of anticancer drugs [3,4,5]. Selective activation of prodrugs can be achieved by intrinsic differences of enzyme expression between tumor and normal tissues [6]. Many designed prodrugs that selectively release active drugs by overexpressed enzymes in the tumor microenvironment, including caspases, cathepsins, and matrix metalloproteinases (MMPs), have greatly increased the safety of chemotherapy with minimal side effects [7,8,9,10]. However, such approaches have been challenged by unfavorable pharmacokinetics (PK), indicating a short in vivo half-life and poor tumor targeting owing to their small molecule structure, resulting in limited antitumor efficacy [11].

Albumin is the most abundant protein in the blood and has 17 disulphide bonds with one free thiol from unpaired cysteine (Cys34), which has emerged as a versatile protein carrier to improve the PK profile of anticancer drugs for tumor targeting [12]. The underlying mechanism of albumin-based drug delivery is that anticancer drugs containing thiol-reactive molecules selectively bind to accessible free thiol on Cys34 of endogenous albumins, and thus enhance the half-lives of drugs [13]. This long half-life is attributable mainly to its macromolecular size, being above the kidney filtration threshold, as well as receptor-mediated salvage mechanism, preventing degradation, facilitated by the neonatal Fc receptor (FcRn) [14]. Therefore, anticancer drugs bound to albumin accumulate within tumors via the enhanced permeability and retention (EPR) effect that is shown in the macromolecular complex by the increased vascular permeability and low lymphatic drainage [15,16]. In clinics, albumin-bound doxorubicin, Aldoxorubicin, has shown potent antitumor efficacy with significantly prolonged patient survival [17]. However, the delivery efficiency to tumors of even the effective drug carriers was, unexpectedly, found to be less than 1~3% in many preclinical studies [18]. This means that a considerable amount of drugs inevitably localized in the off-target tissues and blood stream, which can increase the risk of systemic toxicity. Thus, albumin-based drug delivery of aldoxorubicin still indicated representative side effects of chemotherapy in patients at various stages [19].

## 2. Materials and Methods

### 2.1. Materials

*Phe-Arg-Arg-Gly* (NH_2_-FRRG-COOH) peptide was synthesized from Peptron (Daejeon, Republic of Korea). Cathepsin B-inhibitory siRNA, γ-maleimidobutyric acid, and Monoclonal anti-mouse cathepsin B antibody were purchased from Santa Cruz Biotechnology (Dallas, TX, USA). Cell counting kit-8 (CCK-8) was purchased from Vitascientific (Beltsville, MD, USA). TUNEL assays kit, recombinant cathepsin B, cathepsin E, cathepsin D, caspase-9, and caspase-3 were purchased from R&D systems (Minneapolis, MN, USA). BCA protein quantification kit was purchased from Thermo Fisher scientific (Oakville, ON, USA). Maleimide-PEG_2_-NHS, human serum albumin (HSA), mouse serum albumin (MSA), bovine serum albumin (BSA), hematoxylin and eosin (H&E) staining kit, and doxorubicin hydrochloride were purchased from Sigma Aldrich (Oakville, ON, USA). Fetal bovine serum (FBS), RPMI 1640 medium, Dulbecco’s modified Eagle medium (DMEM) high glucose medium, streptomycin, and penicillin were purchased from WELGENE Inc. (Daegu, Korea). Anti-β-actin antibody was purchased from Abcam (Hanam, Republic of Korea). MDA-MB231 (human breast cancer cells) and H9C2 (rat BDIX heart myoblasts) cell lines were purchased from American Type Culture Collection (ATCC; Manassas, VA, USA). Six-week-old female Balb/c nude mice were purchased from NaraBio, Inc. (Seoul, Korea).

### 2.2. Synthesis of Cathepsin B-Overexpressed Tumor Cell Activatable Albumin-Binding Doxorubicin Prodrug (Al-ProD)

Al-ProD was synthesized via a two-step reaction. At first, maleimide-PEG_2_-NHS (100 mg, 1 equiv) was reacted with NH_2_-FRRG-COOH (251.4 mg, 2 equiv) in anhydrous DMF (10 mL) at 37 °C for 12 h, and maleimide-PEG_2_-FRRG-COOH was purified using HPLC. Second, subsequent synthesis of maleimide-PEG_2_-FRRG-DOX (Al-ProD) was performed by dissolving maleimide-PEG_2_-FRRG-COOH (150 mg, 2 equiv), doxorubicin (DOX; 48.2 mg, 1 equiv), EDC (44.1 mg, 4 equiv), and NHS (40.9 mg, 4 equiv) in anhydrous 10 mL DMF, while stirring at 37 °C for 24 h. Then, Al-ProD was further purified via HPLC, and lyophilized at −90 °C to obtain a red powder (Freeze Dryer, ilShinBioBase, Republic of Korea). After preparation, successful synthesis of Al-ProD was confirmed by measuring purity and molecular weight via HPLC and MALDI-TOF mass spectrometer, respectively.

### 2.3. Characterization of Al-ProD

The albumin-binding property of Al-ProD was firstly evaluated. Briefly, to a human serum albumin (HSA), mouse serum albumin (MSA), or bovine serum albumin (BSA; 700 µM in PBS; pH 7.4), Al-ProD (100 µM) was added and incubated for 0, 5, and 60 min at room temperature. As a control, the HSA solution was pre-incubated with γ-maleimidobutyric acid for 1 h before adding Al-ProD. After incubation, samples were analyzed via native 12% SDS-PAGE gel. The gels were observed by trans-UV using the iBright^TM^ Imaging System (Invitrogen by Thermo Fisher Scientiric), and then stained with coomassie blue for visualizing proteins. The albumin-binding property of Al-ProD was further analyzed using reverse-phased high performance liquid chromatography (RP-HPLC; Agilent cary 300; Agilent Technologies) with ACN/H_2_O gradient from 80:20 to 20:80 for 30 min under a fluorescence detector (Ex/Em: 530/590 nm). In addition, mass shift after incubation of HSA with Al-ProD for 5 min was confirmed by a matrix-assisted laser desorption/ionization time of flight (MALDI-TOF, AB Sciex TOF/TOF 5800 System, Annapolis, MD, USA) mass spectrometer with a cyano-4-hydroycinnamic acid (CHCA) matrix. Next, cathepsin B-specific cleavage of Al-ProD that was pre-incubated with HSA for 5 min (HSA-bound Al-ProD; 10 µM) was assessed by incubating with cathepsin B, cathepsin E, cathepsin D, cathepsin L, caspase-9, or caspase-3 (50 µg) at 37 °C for 24 h, followed by an analysis using HPLC with ACN/H_2_O gradient from 20:80 to 80:20 for 30 min.

### 2.4. Cellular Uptake

To assess intracellular behavior of Al-ProD via fluorescence imaging, 3 × 10^5^ MDA-MB231 and H9C2 cells were seeded in confocal dishes. After 24 h stabilization, each cell was incubated with free DOX or HSA-bound Al-ProD (2 µM) for 48 h at 37 °C. As a control, MDA-MB231 cells were pre-incubated for 2 h with cathepsin B-inhibitory siRNA that was pre-incubated with Lipofectamine 2000 for 40 min at room temperature. Then, cells were washed twice with DPBS, fixed with 5% paraformaldehyde for 15 min, and stained with 4′,6-diamidino-2-phenylindole (DAPI) for 10 min. Fluorescence imaging was performed using a Leica TCS SP8 confocal laser-scanning microscope (Leica Microsystems GmbH; Wetzlar, Germany). The DOX fluorescence in images was quantitatively analyzed using an Image Pro software (Media Cybernetic, Rockville, MD, USA).

### 2.5. Cytotoxicity Assay

The cytotoxicity of Al-ProD was assessed via cell counting kit-8 (CCK) assays. First, 5 × 10^3^ MDA-MB231 or H9C2 cells were seeded in 96-well cell culture plates. After 24 h stabilization, the free DOX or HSA-bound Al-ProD were added to each well and incubated for 48 h. Then, the cells were additionally incubated with culture medium containing 10% CCK solution for 30 min. The cell viability was measured using a microplate reader (VERSAmaxTM; Molecular Devices Corp., San Jose, CA, USA) with 450 nm of wavelength.

### 2.6. Western Blot

Cathepsin B expression in MDA-MB231 and H9C2 cells was analyzed via Western blot [20]. Briefly, 2 × 10^5^ MDA-MB231 or H9C2 cells were seeded in six-well cell culture plates. After 24 h incubation, MDA-MB231 and H9C2 cells were solubilized using lysis buffer including 1% protease inhibitors, and the resulting lysates were centrifuged at 3000 rpm for 40 min to remove debris. The proteins in lysates were quantified by BCA protein quantification kit, and then separated using sodium dodecyl sulfate-polyacrylamide (SDS-PAGE) gel electrophoresis and transferred onto PVDF membranes. Then, membranes were incubated with TBS-T containing 5% bovine serum albumin (BSA) for 1 h to block non-specific IgG binding and incubated with anti-cathepsin B primary antibody for 12 h at 4 °C. Finally, membranes were incubated with HRP-conjugated anti-mouse IgG antibody for 2 h at room temperature and immunoreactive bands were observed via an enhanced chemiluminescence (ECL) system.

### 2.7. Pharmacokinetics (PK)

Mice were bred under pathogen-free conditions at the Korea Institute of Science and Technology (KIST). All experiments with live animals were performed in compliance with the relevant laws and institutional guidelines of Institutional Animal Care and Use Committee (IACUC) in Korea Institute of Science and Technology (KIST), and IACUC approved the experiment (approved number of 2020-123). To assess pharmacokinetic (PK) profiles in vivo, BALB/c nude mice were intravenously injected with free DOX (3 mg/kg) or Al-ProD (3 mg/kg based on DOX contents), and blood samples were collected from mice at pre-determined times (0, 3 h, 6 h, 9 h, 12 h, 24 h, 48 h, 72 h, 96 h, 120 h, and 144 h). Then, each drug in the blood samples was extracted with DMSO by intense vortex and the samples were centrifuged at 2000 rpm for 40 min to obtain as a blood plasma. Finally, amount of free DOX and Al-ProD in samples was analyzed by IVIS Lumina Series III system (PerkinElmer; Waltham, MA, USA).

### 2.8. Biodistribution in Breast Tumor Models

The biodistribution of Al-ProD was assessed in breast tumor models, which were prepared by subcutaneous inoculation of 1 × 10^7^ MBA-MB231 cells into the left flank of BALB/c nude mice. When the tumor volumes were approximately 200–250 mm^3^, the mice were intravenously injected with free DOX (3 mg/kg) or Al-ProD (3 mg/kg based on DOX contents). Then, noninvasive near-infrared fluorescence (NIRF) imaging was performed using an IVIS Lumina Series III system after 0 h, 3 h, 6 h, 12 h, 24 h, 48 h, and 72 h of injection. Fluorescence intensities in tumor regions were quantified via Living Image software. Mice were sacrificed after 12 h of injection for ex vivo imaging, followed by the collection of lung, liver, kidney, spleen, heart, and tumor tissues. Tumor tissues were also cut into 10 µm thick sections for histological assays. Slide-mounted tumor sections were analyzed by Leica TCS SP8 confocal laser-scanning microscope.

### 2.9. Antitumor Efficacy and Toxicity Evaluation

To evaluate the antitumor efficacy, MDA-MB231 tumor-bearing mice were randomly divided into three groups: (i) saline; (ii) free DOX; and (iii) Al-ProD. Then, mice were treated once every three days with free DOX (3 mg/kg) or Al-ProD (3 mg/kg based on DOX contents), at which time tumor volumes were approximately 60–80 mm^3^. Antitumor efficacy was assessed by measuring tumor volumes once every two days, calculated as largest diameter × smallest diameter^2^ × 0.53, once every 2 days. The body weights of mice were also measured once every two days to assess in vivo toxicity. The in vivo toxicity of Al-ProD was further assessed by histological analyses. At 20 days after treatment, major organs were collected from mice and samples were stained with H&E following the manufacturer’s protocol. Then, organ sections were observed using an optical microscope.

### 2.10. Statistics

The statistical significance between two groups was analyzed using Student’s t-test. One-way analysis of variance (ANOVA) was performed for comparisons of more than two groups, and multiple comparisons were analyzed using Tukey–Kramer post-hoc test. Survival data were plotted as Kaplan–Meier curves and analyzed using log-rank test. Statistical significance was indicated with an asterisk (* *p* < 0.05, ** *p* < 0.01, and *** *p* < 0.001) in the figures.

### 2.11. Data Availability

All relevant data are available with the article and its Supplementary Information files, or available from the corresponding authors upon reasonable request.

## 3. Results

### 3.1. Albumin-Binding and Selective Activation of Al-ProD

**Figure 1:**
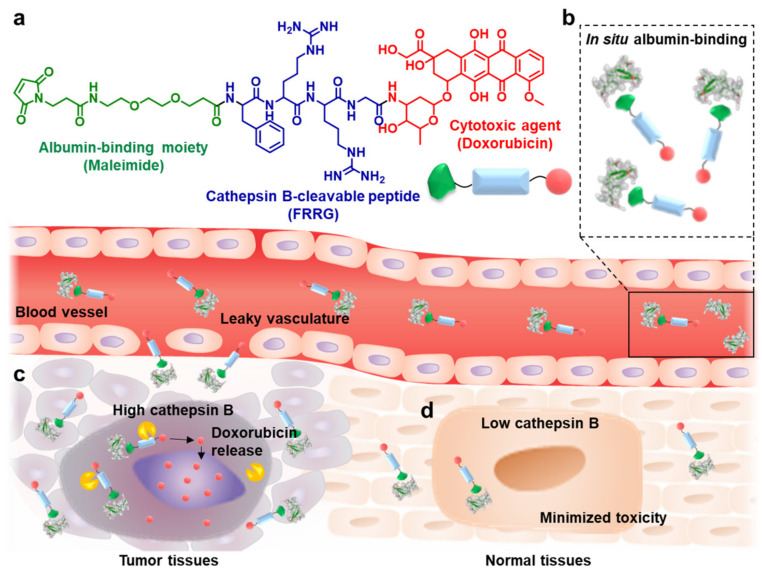
**In situ albumin-mediated cancer-targeted therapy byAl-ProD.** (**a**) The Al-ProD is prepared by conjugating doxorubicin (DOX) to the C-terminus of cathepsin B-cleavable peptide (FRRG) and introducing a maleimide group to the N-terminus of peptide. (**b**) Intravenously injected Al-ProD efficiently binds to in situ circulating albumin in blood vessels. (**c**) Albumin-bound Al-ProD greatly enhances tumor accumulation via albumin-mediated passive tumor targeting and induces a potent antitumor efficacy by selectively releasing free DOX in cathepsin B-overexpressed tumor cells. (**d**) Concurrently, Al-ProD significantly reduced toxicity toward normal tissues with innately low cathepsin B expression by maintaining a non-toxic inactive state, thereby increasing the safety of chemotherapy.

Herein, we propose cathepsin B-overexpressed tumor cell activatable albumin-binding doxorubicin prodrug, Al-ProD, which can effectively deliver anticancer drugs by in situ albumin-mediated passive targeting with minimal side effects. The Al-ProD was prepared by conjugating doxorubicin (DOX) to C-terminus of cathepsin B-cleavable peptide (NH_2_-FRRG-COOH; NH_2_-*Phe-Arg-Arg-Gly*-COOH) and introducing a maleimide group to the N-terminus of peptide (Figure 1a). The maleimide group in the Al-ProD selectively bound to the thiol in physiological pH, thereby allowing the covalent binding with in situ circulating albumin (Figure 1b). Moreover, FRRG peptide is a well-known substrate of cathepsin B, which is associated with tumor invasion and metastasis as a promising cancer biomarker overexpressed in malignant tumors compared with normal tissues in clinical studies [21,22]. Compared with other substrate peptide of cathepsin B, FRRG peptide exhibited high specificity against the target enzyme without non-specific cleavage and, especially, it was reported that G-DOX cleaved from FRRG-DOX by enzymatic cleavage was additionally metabolized into free DOX by intracellular proteases [23,24]. Therefore, the in situ albumin-bound Al-ProD greatly enhances tumor accumulation with prolonged in vivo half-life and induces a potent antitumor efficacy by selectively releasing free DOX in cathepsin B-overexpressed tumor cells (Figure 1c). Concurrently, toxicity toward normal tissues with innately low cathepsin B expression is significantly reduced by maintaining a non-toxic inactive state, thereby increasing the safety of chemotherapy (Figure 1d). In the present study, albumin-binding of Al-ProD was confirmed on human serum albumin (HSA), mouse serum albumin (MSA), and bovine serum albumin (BSA). Selective action of Al-ProD was studied in breast cancer cells and cardiomyocytes, indicating differential levels of cathepsin B. The in vivo pharmacokinetics and tumor regression effect with minimal toxicity were also carried out in breast cancer models.

**Figure 2:**
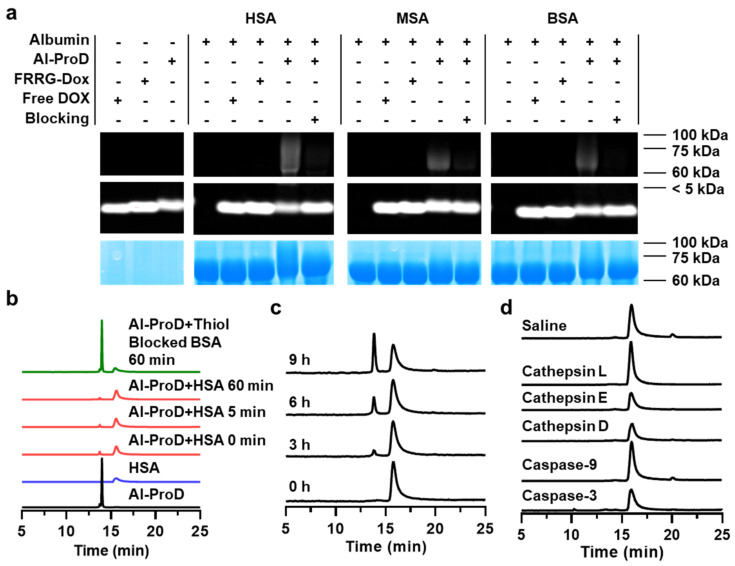
**Albumin binding and selective activation ofAl-ProD.** (**a**,**b**) Albumin-binding of Al-ProD. Al-ProD was incubated with human serum albumin (HSA), mouse serum albumin (MSA), or bovine serum albumin at room temperature. As a control, the HSA solution was pre-incubated with γ-maleimidobutyric acid to block thiol in HSA. In addition, free DOX or FRRG-DOX with the absence of a maleimide group were also incubated with three types of serum albumin. After incubation, samples were analyzed via (**a**) SDS-PAGE gel and (**b**) RP-HPLC. (**c**,**d**) HPLC chromatograms when Al-ProD was incubated with (**c**) cathepsin B or (**d**) other enzymes.

The cathepsin B-overexpressed tumor cell activatable albumin-binding doxorubicin prodrug, Al-ProD, which consists of albumin-binding maleimide group, cathepsin B-cleavable peptide (NH_2_-FRRG-COOH; NH_2_-*Phe-Arg-Arg-Gly*-COOH), and doxorubicin (DOX), was designed for cancer-targeted therapy with minimal side effects. The Al-ProD was synthesized by conjugating DOX to C-terminus of FRRG peptide and introducing a maleimide group to the N-terminus of peptide (Figure S1). After the reaction, 99% of Al-ProD was purified with HPLC (Figure S2). The successful synthesis was also confirmed via matrix-assisted laser desorption ionization time-of-flight (MALDI-TOF) mass spectrometer, wherein the exact molecular weight of Al-ProD was calculated to be 1370.44 Da for C_64_H_83_N_13_O_21_, and measured to be 1370.616 *m*/*z* [M] (Figure S3). First, the plasma albumin-binding ability of Al-ProD was assessed by various in vitro studies. The Al-ProD and albumin from different species of human (human serum albumin; HSA), mouse (mouse serum albumin; MSA), and bovine (bovine serum albumin; BSA) were clearly observed via the doxorubicin absorbance and coomassie blue staining in SDS-PAGE gel, respectively (Figure 2a). Importantly, the band of Al-ProD was detected below 7 kDa, but the band shifted to 50–75 kDa after incubation with HSA, BSA, or MSA for 1 h. MALDI-TOF mass spectrometer further confirmed the molecular weight shift of the HSA from 66,409 to 67,780 *m*/*z* when incubated with Al-ProD, showing a mass difference comparable to that of Al-ProD (1370.616 *m*/*z*), indicating the successful albumin-binding (Figure S4). In contrast, the Al-ProD band was not shifted when each albumin (HSA, MSA, and BSA) was pre-incubated with 4-maleimido butyric acid to block the thiol group. As a control, FRRG-DOX with the absence of a maleimide group and free DOX were also not bound to all types of albumin, only showing the band below 7 kDa. The HSA-binding of Al-ProD was further analyzed by HPLC (Figure 2b). Binding of Al-ProD with HSA in HPLC spectrum was confirmed by a shift of the Al-ProD peak (14 min) to a broad peak at 16 min that appeared to be a free HSA peak, wherein the binding was accomplished within 5 min. However, Al-ProD did not bound to HSA for 60 min when the thiol group of HSA was blocked.

Next, we assessed the cathepsin B-specific cleavage of Al-ProD by incubation with various enzymes. As the Al-ProD releases the DOX molecules via enzymatic degradation in the presence of cathepsin B, it is major of concern whether the cathepsin B can recognize and cleave FRRG peptide without interference by albumin adjacent when Al-ProD was bound to albumin. To address this concern, HSA-bound Al-ProD was incubated with enzyme reaction buffer containing cathepsin B (MES buffer; 50 µg/mL). The result showed that HSA-bound Al-ProD began to be cleaved to glycine-conjugated doxorubicin (G-DOX) after 3 h incubation and G-DOX release was gradually increased for 9 h incubation (Figure 2c). These results were clearly supported by MALDI-TOF analysis, which confirm the molecular weight of G-DOX (calculated mass: 600.58 Da, measured mass: 601.2019 *m*/*z* [M+H], 623.184 [M+Na], and 639.1573 [M+K]) at a newly appeared peak (14 min) after incubation with cathepsin B in the HPLC spectrum (Figure S5). It was already reported that G-DOX cleaved from FRRG-DOX by cathepsin B enzymatic cleavage are efficiently metabolized into free DOX by intracellular proteases [23,24]. In contrast, HSA-bound Al-ProD was not cleaved by other enzymes, such as caspase-3, caspase-9, cathepsin D, cathepsin E, and cathepsin L, or saline (hydrolysis; Figure 2d). These results clearly demonstrate that Al-ProD successfully binds to albumin via a maleimide group and selectively releases DOX molecules in the presence of cathepsin B enzyme.

### 3.2. Cancer Cell-Selective Cytotoxicity of Al-ProD

**Figure 3:**
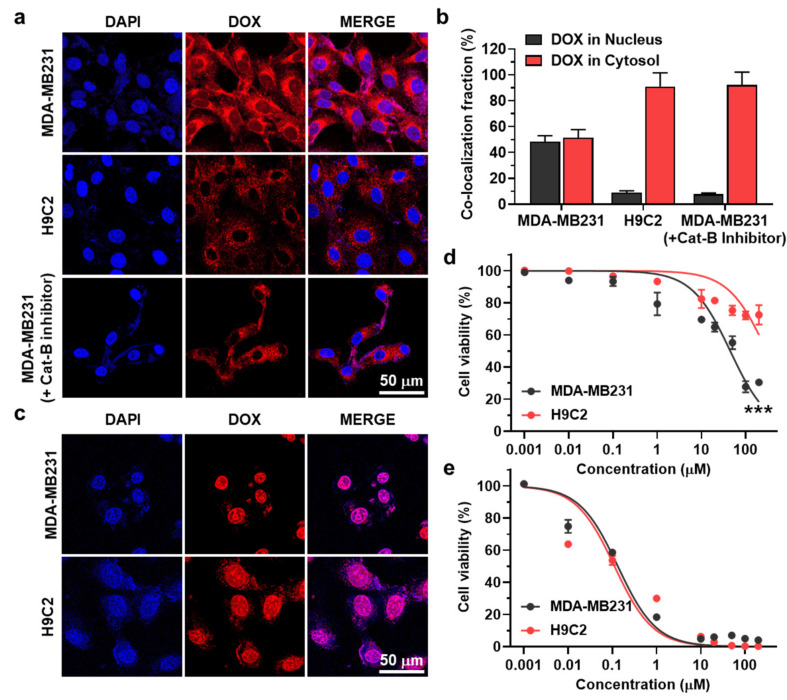
**Cellular uptake and cytotoxicity of Al-ProD.** (**a**) Fluorescence images of MDA-MB231 and H9C2 cells treated with Al-ProD. As a control, MDA-MB231 cells were pre-incubated with cathepsin B-inhibitory siRNA. (**b**) Quantification analysis of DOX fluorescence in nuclei or cytosol of Al-ProD- or free DOX-treated MDA-MB231, H9C2, and cathepsin B-inhibitory siRNA-treated MDA-MB231 cells. (**c**) Fluorescence images of MDA-MB231 and H9C2 cells treated with free DOX. (**d**,**e**) Cytotoxicity of (**d**) Al-ProD or (**e**) free DOX in MDA-MB231 and H9C2 cells. Significance (*** *p* < 0.001) was determined by Student’s *t*-test (**d**).

The in vitro selective activation of Al-ProD premised on differential expression levels of cathepsin B was assessed in breast cancer cells (MDA-MB231) and rat BDIX cardiomyocytes (H9C2). As expected, MDA-MB231 cells expressed a 24.26 ± 3.08-fold high amount of cathepsin B compared with H9C2 cells (Figure S6). Each cell showed a robust uptake of HSA-bound Al-ProD (red color) after 48 h of incubation, as confirmed by confocal laser scanning microscope (CLSM; Figure 3a). However, DOX fluorescence was limited to the cytoplasm of H9C2 cells, whereas that in MDA-MB231 cells was observed in nuclei. The HSA-bound Al-ProD also remained in the cytoplasm of cathepsin B-suppressed MDA-MB231 cells, which are pre-treated with cathepsin B-inhibitory siRNA for 24 h. Quantitatively, DOX fluorescence in nuclei was 2.48–2.89-fold stronger in HSA-bound Al-ProD-treated MDA-MB231 cells than H9C2 and cathepsin B-suppressed MDA-MB231 cells (Figure 3b). In contrast, intracellular free DOX was clearly observed at the nuclei in both MDA-MB231 and H9C2 cells, regardless of cathepsin B expression (Figure 3c). As the mode of action of DOX is intercalation into DNA base pairs, inducing breakage of DNA strand and inhibition of DNA and RNA replication, the DOX moleucle inside the nuclei of cells is an important indicator of its cytotoxicity. As a result, this differential cellular uptake of Al-ProD resulted in cancer cell-selective cytotoxicity [25]. The IC_50_ value of HSA-bound Al-ProD in MDA-MB231 was measured to be 7.33 µM, while it was >200 µM in H9C2 cells after 48 h incubation, which showed about a 30-fold difference that indicates cancer cell-selective cytotoxicity (Figure 3d). In contrast, free DOX exhibitied indiscriminate cytotoxicity in both MDA-MB231 and H9C2 cells with nearly similar IC_50_ values (Figure 3e). These results suggest that Al-ProD can efficiently eradicate cancers with minimal off-target toxicities toward normal tissues by selective activation in cancer cells.

### 3.3. Pharmecokinetics and Tumor Targeting of Al-ProD

**Figure 4:**
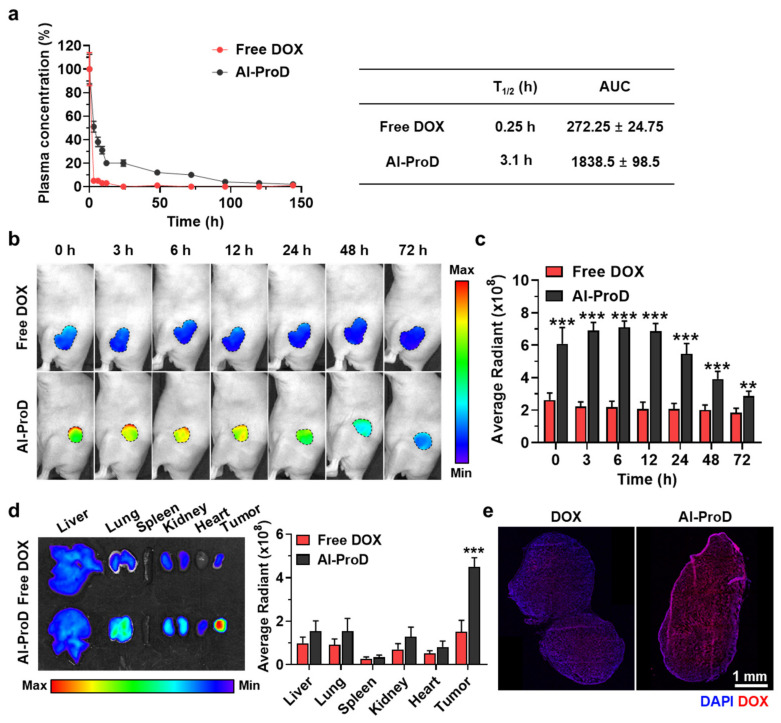
**Pharmacokinetics and biodistribution of Al-ProD.** (**a**) PK profiles of Al-ProD and free DOX. Area under the curve (AUC) was calculated by Origin 2020 software. (**b**) NIRF images of MDA-MB231 tumor-bearing mice treated with Al-ProD of free DOX. (**c**) Quantification analysis on the DOX fluorescence at tumor tissues in NIRF images. (**d**) Ex vivo imaging of organs from mice treated with Al-ProD or free DOX after 12 h injection. (**e**) Quantification analysis of the DOX fluorescence at major organs in ex vivo imaging. (**e**) Fluorescence images of whole tumor tissues after 12 h of Al-ProD or free DOX treatment. Significance (** *p* < 0.01, *** *p* < 0.001) was determined by Student’s *t*-test (**c**,**d**).

To evaluate the high tumor accumulation of albumin-binding Al-ProD by extended in vivo half-life, the pharmacokinetics (PK) of Al-ProD and free DOX was firstly compared in BALB/c nude mice after intravenous injection at a dose of molar equivalent to 3 mg/kg of doxorubicin. In contrast to free DOX, showing fast in vivo clearance with a half-life of 15 min, Al-ProD showed a significantly extended half-life of more than 3 h (Figure 4a). In addition, a detectable amount of the Al-ProD remained for 144 h in the body, indicating the dramatically extended residence time in vivo. As a result, the area under the curve (AUC) of Al-ProD was approximately sevenfold increased compared with that of free DOX. The tumor accumulation of Al-ProD by extended in vivo half-life was further assessed via noninvasive near-infrared fluorescence (NIRF) imaging in breast tumor models. The breast tumor-bearing mice were prepared by subcutaneous inoculation of MDA-MB231 cells (1 × 10^7^) into BALB/c nude mice, and free DOX (3 mg/kg) or Al-ProD (3 mg/kg based on DOX contens) were intravenously injected into mice. In the case of free DOX, the DOX fluorescence in tumor tissues was rapidly decreased for 6 h owing to its rapid in vivo clearance by a short half-life (Figure 4b). However, DOX fluorescence of Al-ProD in tumor tissues was significantly stronger than free DOX at all time points and was retained for 72 h of injection, which indicates high tumor accumulation by albumin-mediated passive targeting effect. The DOX fluorescence from tumor tissues was quantitatively 3.33–4.08-fold stronger in mice treated with Al-ProD than free DOX after 12 h injection (Figure 4c). Ex vivo imaging after 12 h of injection further confirmed the high tumor accumulation of Al-ProD, wherein the DOX fluorescence in tumor tissues was 3.41–4.92-fold stronger than the free DOX group (Figure 4d). Finally, histological analyses also indicated strong DOX fluorescence (red color) in whole tumor tissues from mice treated with Al-ProD compared with free DOX (Figure 4e). In contrast, only a small quantity of free DOX was observed in tumor tissues. These results demonstrate that the abumin-binding property of Al-ProD greatly extended the in vivo half-life of drugs, leading to high tumor accumulation via a passive targeting effect.

### 3.4. Antitumor Efficacy and Toxicity Studies of Al-ProD in Breast Tumor Models

**Figure 5:**
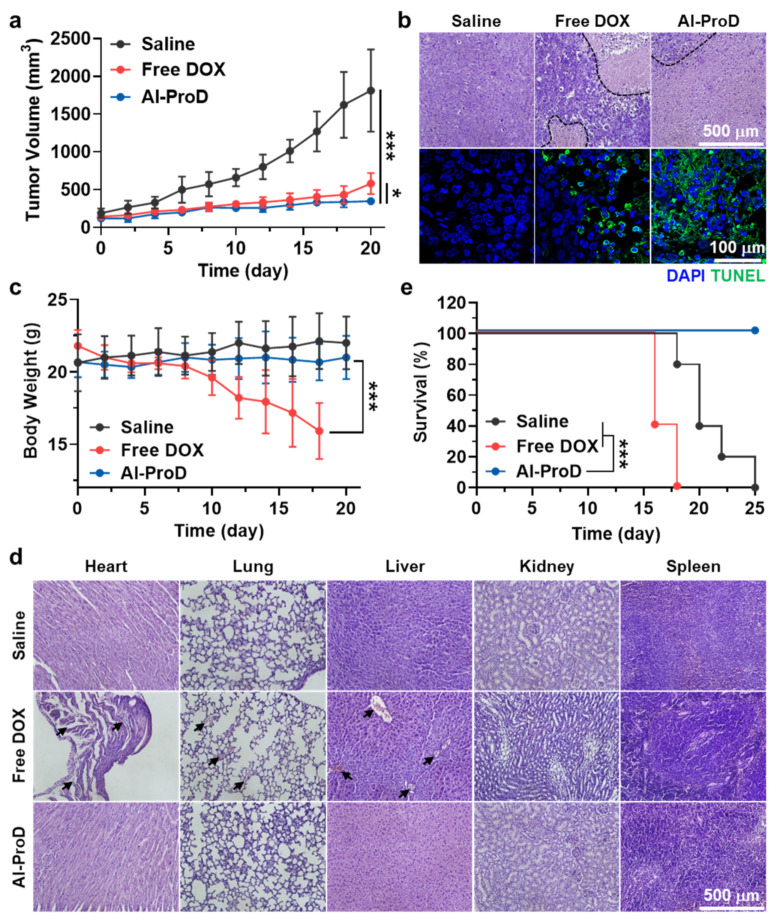
**Antitumor efficacy and toxicity evaluation of Al-ProD.** (**a**) Tumor growth curves of MDA-MB231 tumor-bearing mice after saline, free DOX, or Al-ProD treatment once every three days. (**b**) Tumor tissues stained with H&E or TUNEL to assess antitumor efficacy on day 20 after treatment. (**c**) Body weights during treatment. (**d**) Major organs stained with H&E to assess structural abnormalities on day 20 after treatment. (**e**) Mice survival during treatment. Significance (* *p* < 0.05 and *** *p* < 0.001) was determined by one-way ANOVA with the Tukey−Kramer post-hoc test (**a**,**c**) or log-rank test (**e**).

To evaluate the antitumor efficacy, MDA-MB231 tumor-bearing mice prepared by same protocol as in Figure 4b were treated with free DOX (3 mg/kg) or Al-ProD (3 mg/kg based on DOX contens) once every three days. Importantly, Al-ProD (347.42 ± 25.9 mm^3^) significantly decreased the tumor volume compared with free DOX (580.25 ± 139.92 mm^3^; *p* < 0.05) and saline (1810.98 ± 544.56 mm^3^; *p* < 0.001) groups on day 20 after treatment (Figure 5a). Tumor tissues stained with H&E and TUNEL also showed greatly elevated damaged areas and apoptosis in the Al-ProD group compared with the free DOX and saline groups, which clearly indicated the potent antitumor efficacy by albumin-mediated passive targeting of Al-ProD (Figure 5b). Next, we assessed the reduced off-target toxicity of Al-ProD by high cancer selectivity during treatment. Body weights of mice in the free DOX group gradually decreased during treatment owing to severe systemic toxicity, while those in the Al-ProD group showed no significant body weight loss, similar to the saline group (Figure 5c). Furthermore, normal organs stained with H&E exhibited structural abnormalities in the free DOX group, whereas only negligible toxicity was observed in organs of the Al-ProD group on day 20 after treatment (Figure 5d). In agreement with the above results, the mice in free DOX group were all dead within 18 days, with a median survival of 16 days, whereas Al-ProD-treated mice survived over 25 days (Figure 5e). As a control, median survial of the saline group was measured to be 20 days, wherein the mice were dead as a result of tumor progression. Collectively, our findings demonstrate that Al-ProD effectively inhibits tumor growth without side effects, thereby allowing effective and safe chemotherapy.

## 4. Conclusions

In summary, we proposed achieving the effective and safe chemotherapy with cathepsin B-overexpressed tumor cell activatable albumin-binding doxorubicin prodrug (Al-ProD) via albumin-mediated drug delivery. Al-ProD, which consists of albumin-binding maleimide, cathepsin B-cleavable peptide, and doxorubicin, efficiently bound to plasma albumin, thereby dramatically extending the in vivo half-life of doxorubicin. Importantly, highly accumulated Al-ProD in the tumor tissues via albumin-mediated passive targeting selectively released doxorubicin in cathepsin B-overexpressed cancer cells, which provoked potent antitumor efficacy. Concurrently, Al-ProD significantly reduced the toxicity against normal tissues with innately low cathepsin B by maintaining an inactive state. As a result, localized tumor delivery of doxorubicin by Al-ProD greatly inhibited the breast tumor progression with minimized side effects. Compared with the conventional drug delivery system that encapsultes active drugs into nanoparticles, Al-ProD can prevent the off-target toxicities by accidental drug leakage during circulation. In addition, this system also reduces the risk of potential side effects from carrier materials by using the natural delivery carrier. Finally, presice and consice structures allow the simple preparation protocol, thereby overcoming the fundamental problems of targeted drugs for clinical translation, such as difficulty in quality control (QC) and mass production. Therefore, this study provides a promising approach for effective and safe chemotherapy, which may open new avenues for drug design and provide significant advances for translational nanomedicine. However, unexpectedly low delivery efficiency of targeted drugs is still a common limitation of current drug delivery systems. Thus, many researchers are making efforts to develop the advanced formulation and to increase the understanding of the complex tumor microenvironment that reduces the delivery efficiency of targeted drugs. Considering the clinical success of albumin-mediated drug delivery to improve the pharmacokinetics and tumor targeting of drugs as well as ongoing pipelines like Al-ProD will further move albumin-binding drugs from bench to bedside.

## Data Availability

Not applicable.

## Supplementary Materials

The following are available online at https://www.mdpi.com/article/10.3390/pharmaceutics14010083/s1, Figure S1. Synthetic scheme to prepare cathepsin B-overexpressed tumor cell activatable albumin-binding doxorubicin prodrug (Al-ProD). Figure S2. The purity (>99%) of Al-ProD was confirmed by high performance liquid chromatography (HPLC). Figure S3. The molecular weight of Al-ProD was confirmed via MALDI-TOF mass spectrometer. Figure S4. MALDI-TOF analysis results of human serum albumin and human serum albumin-bound Al-ProD. Figure S5. Metabolite assay of Al-ProD. Glycine-conjugated doxorubicin (G-DOX) release from Al-ProD was confirmed via MALDI-TOF mass spectrometer. Figure S6. Cathepsin B expression levels of MDA-MB231 and H9C2 cells. (left) Western blot analysis of cathepsin B of MDA-MB231 cancer cells and H9C2 normal cells. (right) Relative expression levels of cathepsin B in each cell.
