# Supplementary material for: Cathepsin B-Overexpressed Tumor Cell Activatable Albumin-Binding Doxorubicin Prodrug for Cancer-Targeted Therapy
Source: Pharmaceutics. 2021 Dec 29;14(1):83. doi: 10.3390/pharmaceutics14010083 (PMC8780658; doi:10.3390/pharmaceutics14010083)
Supplement: Supplementary file 1 [file pharmaceutics-14-00083-s001.zip › pharmaceutics-1492921-supplementary.pdf]

pharmaceutics

Supplementary Materials: Cathepsin B-overexpressed Tumor Cell Activatable Albumin-Binding Doxorubicin Prodrug for Cancer-Targeted Therapy

Hanhee Cho, Man Kyu Shim, Suah Yang, Sukyung Song, Yujeong Moon, Jinseong Kim, Youngro Byun, Cheol-Hee Ahn and Kwangmeyung Kim

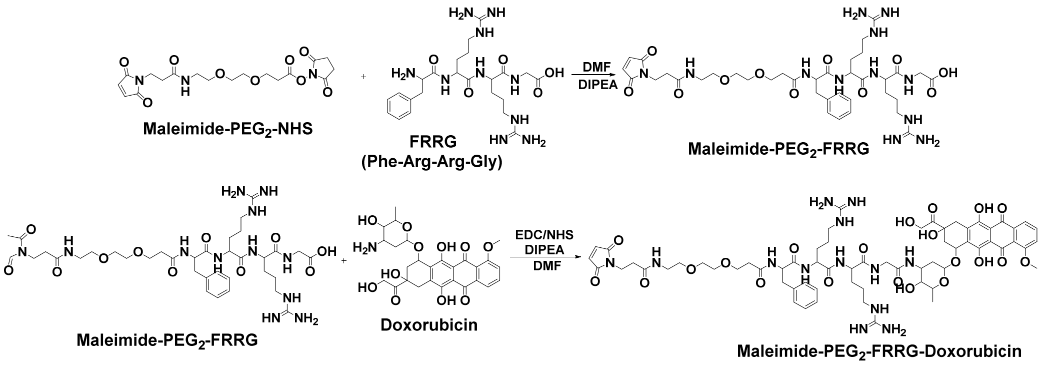

Figure S1. Synthetic scheme to prepare cathepsin B-overexpressed tumor cell activatable albumin-binding doxorubicin prodrug (Al-ProD). First, maleimide-PEG$_2$-NHS (100 mg, 1 equiv) was reacted with NH$_2$-FRRG-COOH (251.4 mg, 2 equiv) in anhydrous DMF (10 mL) at 37 °C for 12 h, and maleimide-PEG$_2$-FRRG-COOH was purified using HPLC. Second, subsequent synthesis of maleimide-PEG$_2$-FRRG-DOX (Al-ProD) was performed by dissolving maleimide-PEG$_2$-FRRG-COOH (150 mg, 2 equiv), doxorubicin (DOX; 48.2 mg, 1 equiv), EDC (44.1 mg, 4 equiv) and NHS (40.9 mg, 4 equiv) in anhydrous 10 mL DMF, while stirring at 37 °C for 24 h. Then, the Al-ProD was further purified via HPLC with equal solvent condition, and lyophilized at −90 °C to obtain as a red powder (Freeze Dryer, ilShinBioBase, Republic of Korea).

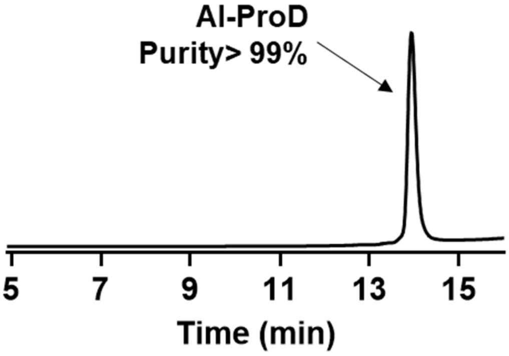

Figure S2. The purity (> 99%) of Al-ProD was confirmed by high performance liquid chromatography (HPLC).

Pharmaceutics **2022**, *14*, 83. https://doi.org/10.3390/pharmaceutics14010083 www.mdpi.com/journal/pharmaceutics

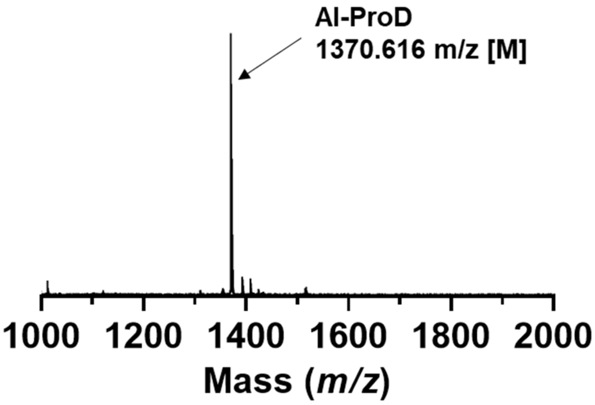

Figure S3. The molecular weight of Al-ProD was confirmed via MALDI-TOF mass spectrometer. The molecular weight of Al-ProD was calculated to be 1370.44 Da for $C_{64}H_{83}N_{13}O_{21}$, and measured to be 1370.616 *m*/*z*.

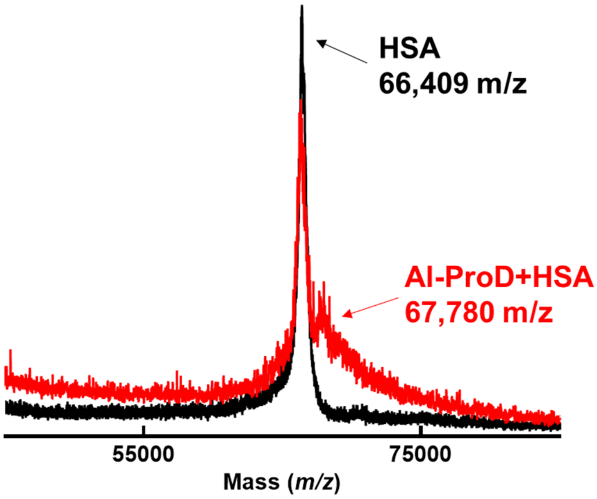

Figure S4. MALDI-TOF analysis results of human serum albumin and human serum albumin-bound Al-ProD.

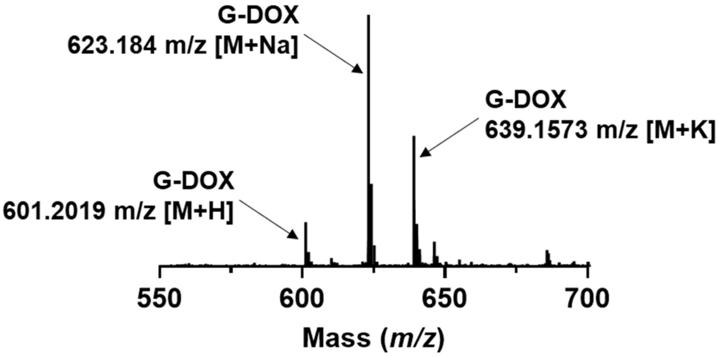

Figure S5. Metabolite assay of Al-ProD. Glycine-conjugated doxorubicin (G-DOX) release from Al-ProD was confirmed via MALDI-TOF mass spectrometer. For this analysis, Al-ProD was incubated with MES buffer containing cathepsin B (50 µg), and enzyme reaction buffer was analyzed. As a result, the molecular weights of G-DOX were measured to be 601.2019 *m*/*z* [M+H], 623.184 [M+Na] and 639.1573 [M+K].

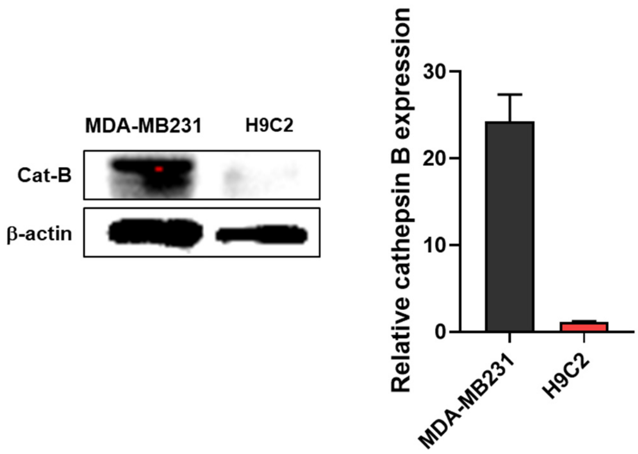

Figure S6. Cathepsin B expression levels of MDA-MB231 and H9C2 cells (left) Western blot analysis of cathepsin B of MDA-MB231 cancer cells and H9C2 normal cells. (right) Relative expression levels of cathepsin B in each cell; MDA-MB231 cells expressed 24.26 ± 3.08-fold higher amount of cathepsin B than H9C2 cells.
